# Adapting emergency care guidance for better outcomes

**DOI:** 10.2471/BLT.24.021024

**Published:** 2024-10-01

## Abstract

A toolkit for emergency care is being adapted for use across a wide range of countries and is having a significant impact on outcomes. Gary Humphreys reports.

Dr Raed Habach does not miss the drama. An emergency clinician and trauma team lead at the Emergency Medicine Institute in Chișinău, Moldova, he vividly remembers the kinds of situation that arose in the emergency department’s red zone prior to the introduction of a new emergency care practice in September 2022.

“It was hectic at times, working to stabilize a patient while summoning specialists to assess the case. Each specialist would enter in turn, calling for different tests and treatments based on their priorities as we worked to save the patient’s life. Without a designated team leader, it was challenging; time was lost, patients too.”

Since June 2023, the institute has been taking a new approach based on the Basic Emergency Care (BEC) course that was developed by the International Committee of the Red Cross and the International Federation for Emergency Medicine, and is part of a broader package addressing emergency care known as the World Health Organization (WHO) Emergency Care Toolkit, an open-access bundle of interventions.

“The BEC course is designed to equip a range of health workers from paramedics and nurses to doctors and specialists with essential skills for emergency care, including early recognition, assessment, treatment, management and referral,” explains Dr Emilie Calvello Hynes, an emergency health-care expert in the clinical services and systems unit at WHO.

The course emphasizes the importance of a systematic approach to initial assessment, early recognition of critical illness and timely intervention to improve patient outcomes, articulated through the “ABCDEs” of emergency care.

The “ABC” stands for Airway, Breathing and Circulation – the three vital functions that must be quickly evaluated and managed to prevent the patient’s deterioration; the “D” and “E” stand for Disability (assessing neurological status) and Exposure/Environment (ensuring the patient is fully exposed to allow assessment for other injuries and managing environmental conditions, such as addressing hypothermia).

The BEC course also emphasizes the importance of a primary and secondary survey, the first addressing immediately life-threatening conditions such as a blocked airway; the second involving a head-to-toe examination to identify injuries or issues that are not immediately life-threatening but require intervention within hours.

“The tools [are] implementable in all settings.”

Emilie Calvello Hynes

“All this information is directed to the trauma team leader, who assesses the situation and decides priorities for the next steps – whether to stabilize the patient, intubate, or focus on specific injuries,” says Habach. “The approach leverages the power of teamwork and ensures that patients are resuscitated, treated and reach definitive care more quickly, greatly improving their chances.”

According to Calvello Hynes, there are indications that use of the toolkit is associated with significantly reduced mortality and morbidity in medical and trauma emergencies, particularly in low- and middle-income countries. These include as-yet-unpublished data on pilots in Nepal, Uganda and Zambia where reductions in deaths ranged from a third to a half. 

The impact on outcomes remains to be seen in Moldova, where a pilot study involving two hospitals began in July; but according to Dr Vitalii Stetsyk, technical officer on clinical management in the WHO Regional Office for Europe, the early indications are good, with over 100 patients with major trauma having already benefitted and a documented decline in emergency care-related mortality.

For Dr Teri Reynolds, head of the WHO clinical services and systems unit, one of the keys to the toolkit’s success is the extent to which it reflects local demand and local resources. “WHO’s Quality Assurance, Norms and Standards team played a key role by designing a normative pathway and setting up a dedicated expert advisory group, but the toolkit was primarily developed in collaboration with health workers and local implementers to ensure that its constituent tools are practical and context-relevant,” she says.

Moldova is a case in point. According to Stetsyk, WHO collaborated with national experts from the start, adapting guidance to make sure that it was practical and implementable. “Initially, WHO provided training to physicians, explaining the goals and intended approach, and introduced protocols considered to be best practice. Together with expert consultants from various hospitals, they then adapted the protocols to reflect local practices and resources,” Stetsyk explains.

WHO has taken the same approach in countries across a range of resource settings. “The foundational thinking is that the sequence of actions we advocate in emergency care can be universally applied, with any adaptations being made according to the resources available,” Stetsyk says. “In settings with limited resources, patient assessment might only involve clinical examinations or basic imaging. However, in more resource-abundant environments, after a full secondary survey we can also implement a full-body computed tomography (CT) scan to thoroughly assess and accurately identify all injuries. This is a protocol that we have incorporated into our guidelines in both Moldova and Ukraine.”

Management of bleeding is another example. In low-resource environments, interventions such as direct pressure, suturing and potentially surgery are employed. In higher-resource settings, more advanced technologies may be used. In Moldova, endovascular treatment is available which involves minimally invasive surgery under X-ray guidance. “The technique allows clinicians to identify and seal bleeding vessels within the pelvis using an expandable air balloon, effectively stopping the bleed,” Habach explains. “This rapid intervention requires skilled personnel, but significantly improves outcomes by providing immediate, localized treatment.”

Calvello Hynes believes that this adaptability has been one of the reasons for the initiative’s widespread success. “WHO created the toolkit to be implementable in all settings,” she says. “We have also designed a comprehensive implementation package that allows for adaptation reflecting local resources.”

WHO has extended its support by conducting specific implementation guidance training to high-level emergency care clinicians in each region (typically focal points from the health ministries), to ensure effective dissemination of the toolkit and to encourage local champions and boost adoption of the toolkit in hospitals.

“The approach leverages the power of teamwork.”

Raed Habach

The adaptability not only allows countries to focus on their assessed needs but makes it possible to use the toolkit for different purposes. “From a normative standpoint, this is really interesting,” Calvello Hynes says, “because you have countries like Afghanistan using it as a part of the humanitarian response to emergencies, while Pan American Health Organization countries such as Costa Rica are using it to improve post-crash care.”

Moldova, like neighbouring Ukraine, is using the toolkit to boost emergency response capacity. The first BEC course in Moldova took place in Chișinău in February 2023. Conducted by six internationally certified Master trainers, the training included interactive and clinical scenario-based discussions, and practical skill stations, allowing participants to develop their skills in medical emergency management according to ABCDE principles and international standards.

To sustain the initiative, WHO has also invested in building national capacity for in-country roll-out of the Basic Emergency Care course through partnerships with the national medical university’s clinical simulation centre, nursing schools and professional societies.

Twelve national trainers have been certified and will be instrumental in the continuous expansion of the course across hospitals in Moldova and abroad. The Ministry of Health is preparing adjustments to the national-level guidance to incorporate the evidence-based BEC training into clinical practice in acute care facilities nationwide, following the positive implementation experiences across the country.

Moldova has already shared its experience in implementing BEC with Ukraine, Kazakhstan and Tajikistan. In 2024, four BEC Master trainers from Ukraine were initially trained in Moldova and, alongside their Moldovan counterparts, helped roll out BEC in Ukraine. Additionally, the team of Moldovan BEC instructors was deployed to Tajikistan in 2023 to introduce BEC in the country, training an initial cohort of 29 health-care providers. According to Statsyk, the similarities between the health systems in these different countries greatly facilitated the transfer of the BEC approach and the benefits were greatly appreciated.

For Habach, the benefits are already clear. The day before he was interviewed for this article, ten hours into his 24-hour shift, a 22-year-old patient was brought in by an ambulance crew. He’d been in a car crash and was securely strapped to a spinal board. Habach called the trauma team to the red zone and immediately started on the ABCDEs.

“We found no visible lesions, but examination by our neurosurgeon revealed that he had lost sensation halfway down his torso. There was some discussion about proceeding with an abdominal CT scan with a contrast agent to check for internal injuries without waiting to confirm kidney function.” With the clock ticking, Habach made the call. “We proceeded with the CT scan, which revealed a severe lesion in the cervical spine,” he says. “Thanks to systematic assessment and early recognition of critical injury, thirty minutes from entering the hospital he was undergoing surgery, hugely improving his chances of a positive outcome. That would not have happened before.”

**Figure:**
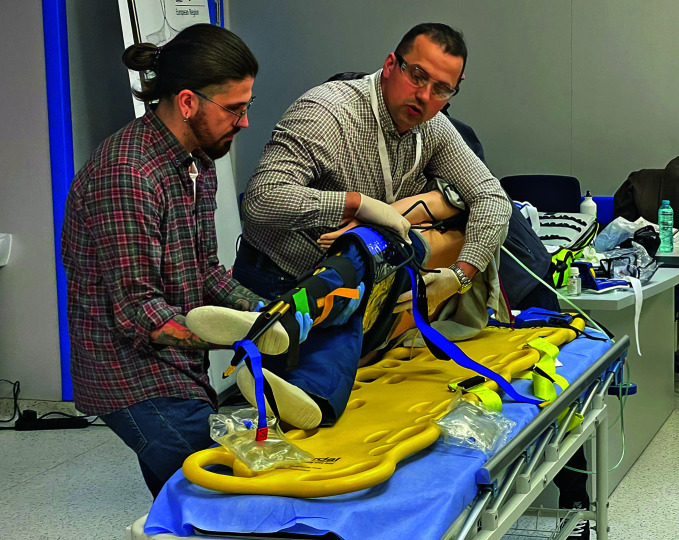
BEC training in Moldova

WHO

